# A Novel Antioxidant Isobenzofuranone Derivative from Fungus *Cephalosporium *sp.AL031

**DOI:** 10.3390/molecules17044219

**Published:** 2012-04-05

**Authors:** Xiang-Zhong Huang, Yun Zhu, Xiao-Li Guan, Kai Tian, Jun-Ming Guo, Hong-Bin Wang, Guang-Miao Fu

**Affiliations:** 1 Key Laboratory of Ethnic Medicine Resource Chemistry, State Ethnic Affairs Commission & Ministry of Education, School of Chemistry and Biotechnology, Yunnan University of Nationalities, Kunming 650500, China; 2 School of Pharmacy, Henan College of Traditional Chinese Medicine, Zhengzhou 450008, China

**Keywords:** *Cephalosporium *sp.AL031 fungus, isobenzofuranone, antioxidant activity

## Abstract

Bioassay-guided fractionation of metabolites from the fungus *Cephalosporium *sp.AL031 isolated from *Sinarundinaria nitida* led to the discovery of a new isobenzofuranone derivative, 4,6-dihydroxy-5-methoxy-7-methylphthalide (**1**), together with three known compounds: 4,5,6-trihydroxy-7-methyl-1,3-dihydroisobenzofuran (**2**), 4,6-dihydroxy-5-methoxy-7-methyl-1,3-dihydroisobenzofuran (**3**) and 4,5,6-trihydroxy-7-methylphthalide (**4**). The structure of the new compound **1** was determined based on MS, 1D and 2D NMR spectral data. Compounds **1**–**4** showed potent antioxidant activity with EC_50_ values of 10, 7, 22 and 5 μM by 1,1-diphenyl-2-picryhydrazyl (DPPH) radical-scavenging assay.

## 1. Introduction

**Figure 1:**
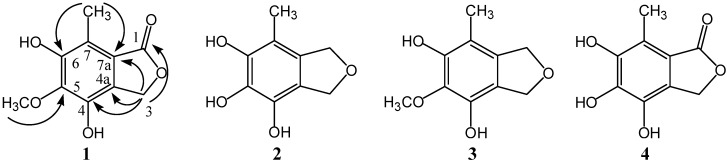
The structures and key HMBC correlations of compounds **1**–**4**.

Isobenzofuranone derivatives are an important class of compounds displaying a variety of biological effects, such as antioxidant, antimicrobial, antiplatelet, cytotoxic activity and antiarrhythmic effects [1,2,3,4]. As sources of bioactive substance, fungi are now considered as efficient producers of biologically active and chemically novel compounds. Some of the more important sources of naturally occurring isobenzofuranone derivatives are fungi [5,6] and higher plants [7]. In our ongoing research on biologically active metabolites from fungi, we investigated the secondary metabolites produced in culture by *Cephalosporium* sp. AL031. This organism is a fungus that was isolated from *Sinarundinaria nitida* grown at Ailao Mountain (Yunnan, China). Previous phytochemical investigation on the fungus revealed a number of phenolic acids [8], dihydroisocoumarin glucosides [9,10] and pyrone derivatives [11,12]. In the present study, we describe the isolation and structure elucidation of a novel isobenzofuranone derivative, namely 4,6-dihydroxy-5-methoxy-7-methylphthalide (**1**), together with three known ones: 4,5,6-trihydroxy-7-methyl-1,3-dihydro-isobenzofuran (**2**), 4,6-dihydroxy-5-methoxy-7-methyl-1,3-dihydroisobenzofuran (**3**) and 4,5,6-trihydroxy-7-methylphthalide (**4**) (Figure 1), from the culture of the fungus *Cephalosporium *sp. AL031. The structure of the new compound **1** was determined based on MS, IR, 1D and 2D NMR spectral data, and the known ones **2**–**4** were identified by comparing their NMR data with those in the literature. The antioxidant activity of compounds **1**–**4** was evaluated by DPPH radical-scavenging assay.

## 2. Results and Discussion

Compound **1** was obtained as colorless needle-like crystals that gave a positive reaction in the 10% H_2_SO_4_-EtOH test. The molecular formula C_10_H_10_O_5_ was deduced from a pseudomolecular ion [M + H]^+^ at *m/z* 211.0628 in the HR-ESI-MS (calcd. for C_10_H_11_O_5_, 211.0607), which was in agreement with the ^1^H- and ^13^C-NMR spectra. The IR spectrum showed characteristic absorption bonds for hydroxyl groups (3365 cm^−1^), saturated methylene (2930 cm^−1^) and a carbonyl group (1685 cm^−1^).

The ^1^H-NMR spectrum of **1**(Table 1) exhibited signals attributable to a methoxyl group at *δ* 3.87 (3H, s), an oxymethylene moiety at *δ* 5.12 (2H, brs) and a methyl group at *δ* 2.42 (3H, s). The ^13^C-NMR and DEPT spectra of **1**(Table 1) also showed the occurrence of three sp^3^-carbon signals related to a methoxyl group (*δ* 59.6), an oxymethylene moiety (*δ* 66.8) and a methyl group (*δ* 8.2). Including one quaternary sp^2^-carbon signal which was attributed to a carbonyl moiety at *δ* 172.7, compound **1** also indicated six quaternary sp^2^-carbon signals at *δ* 125.8, 142.1, 140.8, 149.6, 116.5 and 117.7. The ^1^H-NMR and ^13^C-NMR data was assigned to an isobenzofuranone skeleton.

**Table 1:** ^1^H- and ^13^C-NMR data of **1** and **4** in CD_3_OD. (^1^H at 400 and ^13^C at 100 MHz; *J *in Hz).

Position	Compound 1				Compound 4	
	δ_C_	DEPT	δ_H_	HMBC	δ_C_	δ_H_
1	172.7	C			175.5	
3	66.8	CH_2_	5.12 (2H, brs)	C-1, C-4, C-4a, C-7a	68.9	5.11 (2H, brs)
4	142.1	C			142.3	
4a	125.8	C			128.8	
5	140.8	C			138.9	
6	149.6	C			147.1	
7	117.7	C			119.2	
7 a	116.5	C			114.9	
8	8.2	CH_3_	2.42 (3H, s)	C-6, C-7a	10.6	2.41 (3H, s)
9	59.6	CH_3_	3.87 (3H, s)			

The ^1^H-NMR and ^13^C-NMR data of **1** are very similar to those of 4,5,6-trihydroxy-7-methylphthalide [1] that has been isolated from the fungus *Epicoccum *sp. The only difference between these two compounds is the methoxyl group present in compound **1**. HSQC and HMBC experiments revealed all significant connectivities. The correlation of the methyl group (*δ* 2.42) with C-6 (*δ *149.6) and C-7a (*δ *116.5) in the HMBC spectrum indicated the methyl unit connected to C-7. Similarly, the linkage of the methoxyl moiety at C-5 was deduced from the correlation between the methoxyl group (*δ* 3.87) and C-5 (*δ* 140.8). Furthermore, no correlations were observed between the methyl group (*δ* 2.42) or oxymethylene moiety (*δ* 5.12) and C-5, which unambiguously established the linkage of methoxyl moiety. All NMR data of compound **1** were assigned on the basis of DEPT, HSQC and HMBC experiments. Taken together, compound **1** was determined to be a new isobenzofuranone and named as 4,6-dihydroxy-5-methoxy-7-methylphthalide with the structure shown in Figure 1.

Together with the new compound **1**, three known compounds **2**–**4** were identified by comparison of their spectroscopic data [1,13,14] with published values. We evaluated the antioxidant activity of isolated compounds **1**–**4** by DPPH radical-scavenging assay, which showed potent activity with the EC_50 _values of 10, 7, 22 and 5 μM, respectively. 

## 3. Experimental

### 3.1. General

TLC was preformed with silica gel GF_254_ (Marine Chemical Industry Factory, Qingdao, China), and the spots were visualized by spraying with 10% H_2_SO_4_-EtOH reagent, followed by heating. Column chromatography was performed using silica gel (Marine Chemical Industry Factory, Qingdao, China), reverse-phase C_18_ silica gel (Merck, Germany) and Sephadex LH-20 (Sigma). All reagents were analytical grade and water was distilled-twice. Melting points were measured with an X-4 melting point apparatus and are uncorrected. Optical rotations were recorded on a Perkin-Elmer 241 polarimeter. IR spectra were obtained on a Perkin-Elmer 577 spectrometer with KBr pellets. UV spectra were obtained on a Perkin-Elmer Lambda 900 UV/VIS/NIR spectrophotometer. NMR Spectra were recorded on a Brüker AV-400 spectrometer at 400 MHz ^1^H and 100 MHz for ^13^C in MeOD, and chemical shifts are presented as values relative to tetramethylsilane as an internal standard. Low-resolution electrospray-ionization mass spectrometry (ESI-MS) and HR-ESI-MS were recorded on a Finnigan LCQ-Advantage mass spectrometer and a VG Auto-Spec-3000 mass spectrometer. 

### 3.2. Fungal Material

The fungus *Cephalosporium* sp. AL031 was isolated from *Sinarundinaria nitida* grown at Ailao Mountain, Yunnan, China. It was identified as *Cephalosporium *sp. (strain number AL031), a member of Moniliaceae, by Professor Fa-Rong Yang in the Department of Biology, Yunnan University, and deposited in Key Laboratory of Industrial Microbial Fermentation Engineering of Yunnan Province, China. The isolated fungus was cultured on slants of potato dextrose agar (PDA) at 27 °C for 5 days, and then the fungus was grown on a medium consisting of rice, wheat bran, cornmeal, sugar cane bagasse with a ratio of 12:2:3:3, and incubated for 7 days at 28 °C.

### 3.3. Extraction and Isolation

The fermented material (1.75 Kg) was extracted twice with EtOAc (9.0 L) for 3 days at room temperature, and the extract was evaporated under vacuum. The EtOAc extract was evaluated for antioxidant activity using a DPPH radical-scavenging assay that showed positive effects with an EC_50_ value of 46 µg/mL. A portion of the EtOAc extract (35 g) was subjected to silica gel column chromatography using a stepwise gradient elution of CHCl_3_-MeOH (100:0; 50:1; 20:1; 15:1 and 9:1) to obtain seven fractions (Fr. 1–7). Fr. 1 (5.5 g) was further purified over Sephadex LH-20 column eluted by MeOH, and then recrystallized in CHCl_3_ to give compound **1** (20 mg). Fr. 3 (4.8 g) was rechromatographed over silica gel column eluted by gradient CHCl_3_–MeOH (1:0-10:1) to afford compounds **2** (12 mg). Fr. 4 (3.5 g) was subsequently subjected to Sephadex LH-20 (eluted with MeOH), followed by purification over silica gel column chromatography with CHCl_3_–MeOH (1:0-9:1) as mobile phase to yield compound **3** (15 mg). Compound **4** (25 mg) was collected from Fr. 7 (6.2 g) by silica gel column chromatography (eluted with CHCl_3_–MeOH; 50:1-5:1) and then recrystallized in CHCl_3_.

### 3.4. Characterization of 4,6-Dihydroxy-5-methoxy-7-methylphthalide (***1***)

Colorless needle crystal, M.P. 253–255 °C. UV (MeOH): λmax (log ε_max_): 218 (2.60), 268 (1.02), 308 (0.49) nm. IR (KBr): ν = 3365, 3164, 3021, 2930, 1685, 1611, 1402, 1359, 1286, 1099, 929 cm^−1^. ESI-MS: *m/z* = 211 [M + H]^+^. HR-ESI-MS: *m/z *= 211.0628 (calcd. 211.0607 for C_10_H_11_O_5_, [M + H]^+^). ^1^H- and ^13^C-NMR: see Table 1. 

### 3.5. Bioassay

The antioxidative properties of compounds **1**–**4** were assessed using the 1,1-diphenyl-2-picryl-hydrazyl (DPPH) radical scavenging assay. DPPH is a stable free radical with a strong UV/VIS absorption band at 517 nm. Addition of radical scavenging compounds to a solution of DPPH results in a decrease of absorption. The inhibitory percentage of DPPH was calculated according to the equation as follows: % scavenging activity = [1 − (As/Ac)] × 100; where As and Ac were the absorbance values at 517 nm of the DPPH solutions with sample and without sample, respectively. According to the previous method [15], we evaluated the antioxidant activity of isolated compounds **1**–**4** and the positive control gallic acid by DPPH radical-scavenging assay with the EC_50_ values of 10, 7, 22, 5 and 2 μM, respectively.

## 4. Conclusions

Repeated column chromatography (including normal-phase silica gel and Sephadex LH-20) of the EtOAc extract of the fermentation of the fungus *Cephalosporium* sp. AL031 has led to the isolation of a novel isobenzofuranone derivative, namely 4,6-dihydroxy-5-methoxy-7-methylphthalide (**1**), together with three known ones: 4,5,6-trihydroxy-7-methyl-1,3-dihydroisobenzofuran (**2**), 4,6-dihydroxy-5-methoxy-7-methyl-1,3-dihydroisobenzofuran (**3**), and 4,5,6-trihydroxy-7-methylphthalide (**4**). The structure of the new compound **1** was determined by spectroscopic methods, including 1D NMR, 2D NMR and MS experiments, and the structures of the known compounds including **2**~**4** were identified by comparing their NMR data with those of ones in the literature. All four compounds showed potent anti-oxidative activity by DPPH radical-scavenging assay, which are potential anti-oxidative agents. Comparing the antioxidant activity of compounds **1** and **4**, **3** and **2**, the latter containing three phenolic hydroxyl groups showed potent activity, which indicated the antioxidant activity correlates with the number of hydroxyl groups in the structure.
